# Disease burden and trends of lung cancer attributable to nickel among Chinese and global population: a cross-sectional study

**DOI:** 10.3389/fmed.2024.1497597

**Published:** 2024-12-20

**Authors:** Huaye Lu, Lei Han, Peihong Wu, Xin Liu, Qingtao Jiang

**Affiliations:** ^1^Institute of Occupational Disease Prevention, Jiangsu Provincial Center for Disease Control and Prevention (Jiangsu Provincial Academy of Preventive Medicine), Nanjing, China; ^2^Department of Clinical Medicine, Jiangsu Health Vocational College, Nanjing, China

**Keywords:** global burden of disease (GBD), disability-adjusted life years (DALY), nickel, lung cancer, prediction

## Abstract

**Background:**

Nickel is a well-established carcinogen, and China stands as a significant producer of nickel compounds. Nickel-associated lung cancer is increasingly acknowledged as a pressing public health concern. This study presents a comprehensive analysis at temporal, spatial, and population levels utilizing the most recent data from GBD 2019 to estimate the disease burden of nickel-associated lung cancer from 1990 to 2019, and make predictions to 2035.

**Methods:**

We delineated data on nickel-associated lung cancer concerning mortality, disability-adjusted life years (DALY), and age-standardized rates (ASRs) over a 30-year period based on the global burden of disease (GBD) 2019. Joinpoint regression analysis was utilized to identify temporal changes and to estimate the annual percentage change (APC) as well as the average annual percentage change (AAPC) for each trend segment. The Nordpred model was employed to elaborate on ASRs trends from 1990 to 2019, along with projections for the subsequent 15 years.

**Results:**

In both China and globally, the mortality rate from nickel-associated lung cancer and the associated DALYs have increased by 145.8, 77.8, 120.2, and 64.6%, respectively. ASRs within Chinese and global populations exhibit a pattern characterized by an initial increase followed by a decrease as age progresses, with males higher than females. The trend for DALY indicates an initial rise followed by a decline, peaking in the year 2027.

**Conclusion:**

The age structure of nickel-associated lung cancer patients shows an aging trend, and the ASDR in the Chinese population indicates a potential upward trend when projecting the disease burden of nickel-associated lung cancer over the next 15 years. We should place greater emphasis on the implementation of preventive strategies and the enhancement of the quality of life for current sufferers.

## 1 Introduction

Nickel is a silver-white, ferromagnetic, corrosion-resistant metal element that is widely distributed in the lungs, liver, kidneys, and other tissues and organs. It is also one of the essential trace elements in the human body. A deficiency of nickel can lead to a decrease in enzyme activity and can impact human growth and development (1). However, excessive exposure to nickel can pose health hazards such as neurasthenic reactions, inflammatory responses, and oxidative stress injuries (2, 3). Studies have indicated that occupational populations exposed to high levels of nickel for prolonged periods are at an increased risk of developing lung cancer, nasopharyngeal cancer, and occupational asthma (4–10).

In 1990, the International Agency for Research on Cancer (IARC) classified nickel compounds as Group I carcinogens and metal nickel as a Group II B carcinogen. Na et al. (11) conducted a retrospective cohort study on four nickel-receiving factories and mines, revealing an increased risk of lung cancer death among workers in nickel refining and smelting. Pesch et al. (12) carried out a case-control study within a German occupational population involved in nickel welding, finding a dose-response relationship between nickel exposure and the risk of lung cancer. The mechanism by which nickel exposure induces lung cancer has also been verified. Several studies have demonstrated that molecular mechanisms such as apoptosis and autophagy are induced by nickel exposure (13–16). Other studies have found that epigenetic changes, activation of hypoxia signaling pathways, and production of reactive oxygen species are also implicated in how nickel and its compounds induce cancer at a molecular level (17).

Disability-adjusted life years (DALY) serves as an indicator of the disease burden, calculating all healthy years of life lost from the onset to death. This includes years of life lost due to premature death (YLL) and years of healthy life lost due to disability from illness (YLD). The global burden of disease (GBD) database has been in operation since 1988, its purpose is to analyze the global burden of disease, encompassing health burdens related to disease, risk factors, disabilities, and mortality (18).

China is a leading global producer of nickel compounds, according to the 2022 report on the prevalence of malignant tumors in China, lung cancer has the highest incidence among all malignant tumors (19). However, there is a lack of analysis on the burden of lung cancer related to nickel exposure. Therefore, this paper aims to extract mortality and disease burden data related to nickel-induced lung cancer from the GBD database. The study will analyze the disease burden of nickel-related lung cancer from 1990 to 2019 and its changing trend. Additionally, relevant software will be used to predict the disease burden of nickel-related lung cancer over the next 15 years. The findings aim to provide suggestions for developing occupational disease prevention measures and improving the quality of life for current patients.

## 2 Materials and methods

### 2.1 Study data

Data from this study is obtained from the GBD2019 database and the global health data exchange (GHDx) website,^1^ which provides access to public data. The Institute for Health Metrics and Evaluation (IHME) systematically estimates the incidence, prevalence, mortality, DALY, YLL, and YLD for 369 diseases and injuries in 204 countries and territories (20, 21). The cancer codes used in the GBD correspond to the International Statistical Classification of Diseases and Related Health Problems (Tenth Revision) (ICD-10) (22).

### 2.2 Statistical analysis

In this study, the number of deaths and standardized mortality were chosen to reflect the risk of death in a specific population during a certain period. YLL represents the years of life lost, while YLD reflects years lived with disability. DALY was utilized as an indicator of disease burden for nickel-associated lung cancer, comprehensively reflecting the impact of life loss caused by both YLL and YLD.

ASR stands for the age-standardized rate, while ASMR refers to the age-standardized mortality rate and ASDR represents the age-standardized DALY rate. The 95% uncertainty interval (UIs) was employed to estimate the number of deaths and other indicators, while 100% annual change (APC) and average annual change percentage (AAPC) were used to analyze trends in statistical indicators such as ASDR (abbreviations are utilized following the text). Joinpoint software was utilized for calculating and statistically analyzing AAPC, with R software being used for data cleaning, visual display, and testing at a significance level α = 0.05.

## 3 Results

### 3.1 Mortality and DALY in nickel-associated lung cancer in China and globally

**FIGURE 1:**
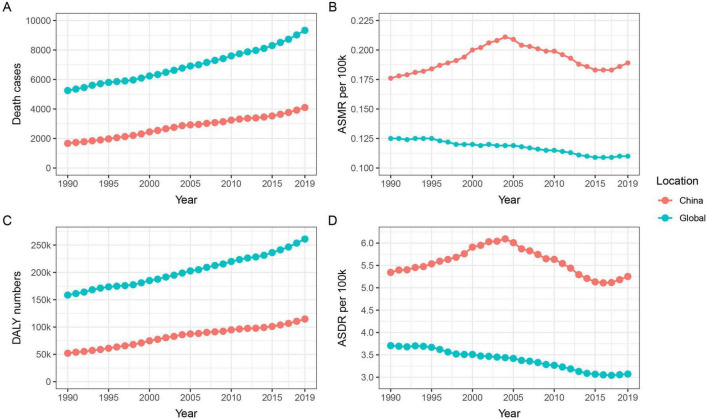
Trends in mortality and DALY for nickel-associated lung cancer in China and globally from 1990 to 2019. **(A)** Temporal trends of the cases of deaths; **(B)** Temporal trends of ASMR; **(C)** Temporal trends of DALY; **(D)** Temporal trends of ASDR.

**FIGURE 2:**
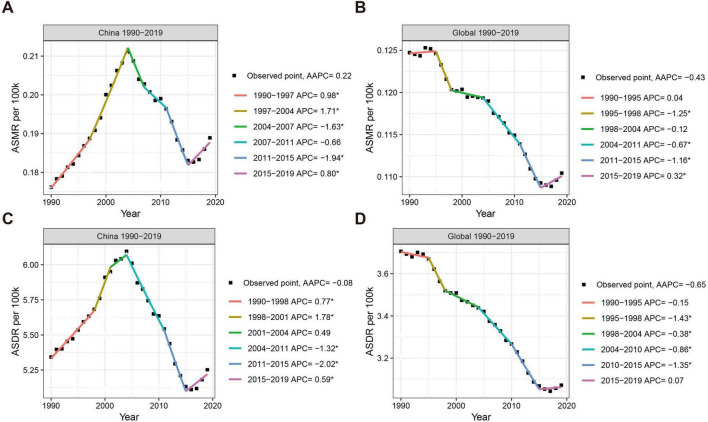
Trends in ASMR and ASDR for nickel-associated lung cancer in China and globally from 1990 to 2019. **(A)** Temporal trends of ASMR and average annual change percentage in China; **(B)** Temporal trends of ASMR and average annual change percentage in global; **(C)** Temporal trends of ASDR and average annual change percentage in China; **(D)** Temporal trends of ASDR and average annual change percentage in global.

We conducted an analysis of the trends in nickel-associated lung cancer deaths and DALY in China and globally from 1990 to 2019. During this period, the total number of nickel-associated lung cancer deaths in China was 83,720, accounting for 40.2% of the global nickel-associated lung cancer deaths. The death rate of nickel-associated lung cancer in China increased by 145.8%, rising from 1,668 to 4,100. Globally, nickel-associated lung cancer deaths increased from 5,248 to 9,330, representing a rise of 77.8% (Table 1 and Figures 1A, B). Furthermore, the DALY caused by nickel-associated lung cancer in China increased from 52,043 person-years to 114,606 person-years, marking an increase of 120.2%. The global DALY due to nickel-associated lung cancer increased from 158,519 person-years to 260,986 person-years, representing a significant increase of 64.6% (Table 1 and Figures 1C, D). As depicted in Figure 2, the ASMR of nickel-associated lung cancer in the Chinese population showed an initial increase followed by a decrease during the period of 1990–2019 (AAPC = 0.22%, *P* < 0.05, Figure 2A). Conversely, there was an overall downward trend globally (AAPC = −0.43%, *P* < 0.05, Figure 2B). The ASDR of nickel-associated lung cancer in China exhibited an initial increase followed by a decrease with no statistical significance (AAPC = −0.08%, *P* > 0.05, Figure 2C), while there was an overall downward trend globally (AAPC = −0.65%, *P* < 0.05, Figure 2D).

**TABLE 1:** Mortality and DALY in nickel-associated lung cancer in China and globally from 1990 to 2019.

Location	Year	Death cases	ASMR (×1/10^5^)	DALY numbers	ASDR (×1/10^5^)
China	1990	1,668	0.176	52,043	3.706
	1995	1,973	0.184	74,810	5.344
	2000	2,445	0.200	61,151	3.509
	2005	2,925	0.209	87,456	6.010
	2010	3,244	0.199	94,825	5.636
	2015	3,528	0.183	101,048	5.535
	2019	4,100	0.189	114,606	3.669
	Change rate (%)	145.8	7.4	120.2	−1.0
	AAPC (%)		0.2 (0.0∼0.4)		−0.1 (−0.3∼0.1)
	*T*		2.2		−0.7
	*P*		**0.029**		0.461
Global	1990	5,248	0.125	158,519	5.910
	1995	5,800	0.125	185,066	3.421
	2000	6,239	0.120	173,493	3.267
	2005	6,913	0.119	202,693	5.132
	2010	7,597	0.115	219,978	3.067
	2015	8,299	0.109	236,227	5.253
	2019	9,330	0.110	260,986	3.071
	Change rate (%)	77.8	−12.0	64.6	−48.0
	AAPC (%)		−0.4 (−0.5∼−0.3)		−0.7 (−0.8∼−0.6)
	*T*		−7.4		−12.1
	*P*		**0.000**		**0.000**

The bold value signifies that the *P*-value is statistically significant.

### 3.2 Analysis of the disease burden caused by lung cancer attributable to nickel exposure

**FIGURE 3:**
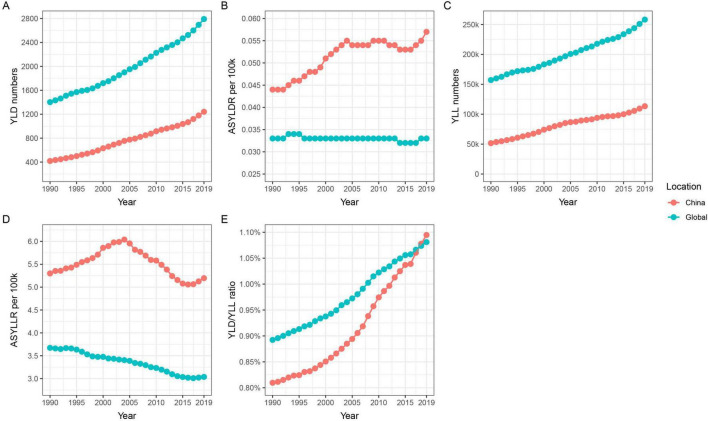
Trends in YLD, YLL, ASYLDR, ASYLLR, and YLD/YLL for nickel-associated lung cancer in China and globally from 1990 to 2019. **(A)** Temporal trends of YLD; **(B)** Temporal trends of ASYLDR; **(C)** Temporal trends of YLL; **(D)** Temporal trends of ASYLLR; **(E)** Temporal trends of YLD/YLL.

From an alternative perspective, the YLL and YLD caused by nickel-associated lung cancer in the Chinese population increased from 51,625 person-years and 418 person-years to 113,365 person-years and 1,241 person-years, respectively, between 1990 and 2019 (Table 2 and Figure 3A, C). The global population saw an increase from 157,118 and 1,402 to 258,195 and 2,790 respectively. It is worth noting that the growth rate of China was higher than that of the world. The age-standardized YLD rate (ASYLDR) increased from 0.044/100,000 to 0.057/100,000 with AAPC = 0.9% (*P* < 0.001), while the global AAPC decreased from 0.033/100,000 to 0.033/100,000 with AAPC = −0.0% (*P* = 0.737) (Table 2 and Figure 3B). The age-standardized YLL rate (ASYLLR) decreased from 5.300/100,000 in 1990 to 5.196/100,000 in 2019, with an AAPC of −0.1% (*P* = 0.408). The global AAPC also showed a decrease from 3.673/100,000 to 3.037/100,000, with an AAPC of −0.7% (*P* < 0.001) (Table 2 and Figure 3D). The increase in YLD/YLL for nickel-associated lung cancer in the Chinese population (35.1%) was higher than that in the global population (21.2%), and by 2018 the YLD/YLL in the Chinese population had surpassed that of the global population for the first time (Table 2 and Figure 3E).

**TABLE 2:** YLD and YLL of lung cancer attributable to nickel from 1990 to 2019.

Location	Year	YLD (person-years)	ASYLDR (×1/10^5^)	YLL (person-years)	ASYLLR (×1/10^5^)	YLD/YLL (%)
China	1990	418	0.044	51,625	5.300	0.810
	1995	500	0.046	60,652	5.489	0.824
	2000	631	0.051	74,179	5.859	0.851
	2005	775	0.054	86,681	5.956	0.894
	2010	915	0.055	93,910	5.581	0.974
	2015	1,037	0.053	100,011	5.078	1.037
	2019	1,241	0.057	113,365	5.196	1.095
	Change rate (%)	196.9	29.5	119.6	−2.0	35.2
	AAPC (%)		0.9 (0.7∼1.1)		−0.1 (−0.3∼0.1)	
	*T*		9.3		−0.8	
	*P*		**0.000**		0.4	
Global	1990	1,402	0.033	157,118	3.673	0.892
	1995	1,570	0.034	171,923	3.635	0.913
	2000	1,719	0.033	183,346	3.476	0.938
	2005	1,952	0.033	200,741	3.388	0.972
	2010	2,226	0.033	217,751	3.233	1.022
	2015	2,468	0.032	233,759	3.035	1.056
	2019	2,791	0.033	258,195	3.038	1.081
	Change rate (%)	99.1	0.0	64.3	−17.3	21.2
	AAPC (%)		−0.0 (−0.1∼0.1)		−0.7 (−0.8∼−0.6)	
	*T*		−0.3		−12.5	
	*P*		0.7		**0.000**	

The bold value signifies that the *P*-value is statistically significant.

### 3.3 Trends in the global burden of disease by region

**FIGURE 4:**
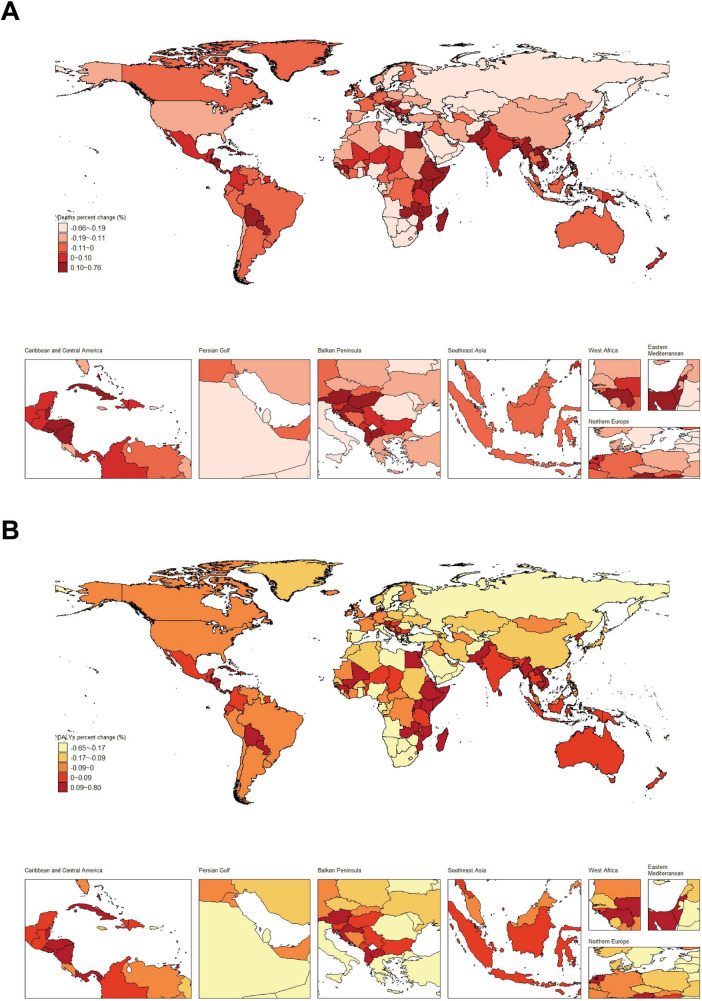
Changes in the proportion of nickel attributable to lung cancer deaths and DALY from 1990 to 2019. **(A)** Proportional changes in nickel-associated lung cancer mortality; **(B)** Trends in nickel attribution for DALY associated with lung cancer.

From a global perspective, there is significant variation in the changes in the burden of nickel-associated lung cancer disease from 1990 to 2019 across different countries. The proportion of lung cancer deaths attributable to nickel has seen substantial increases in Egypt, Pakistan, and Ethiopia, ranging from 0.10 to 0.76%. Conversely, countries such as Russia, Sweden, and Saudi Arabia have experienced notable declines in the nickel attributable proportion of lung cancer deaths, ranging from 0.19 to 0.66% (Figure 4A). When considering the nickel attribution ratio in DALY caused by lung cancer, it is evident that Egypt, Pakistan, Vietnam and other countries have also witnessed significant increases ranging from 0.09 to 0.80%. On the other hand, Russia, Sweden and Saudi Arabia stand out for experiencing the highest declines in this ratio ranging from 0.17 to 0.65% (Figure 4B).

**FIGURE 5:**
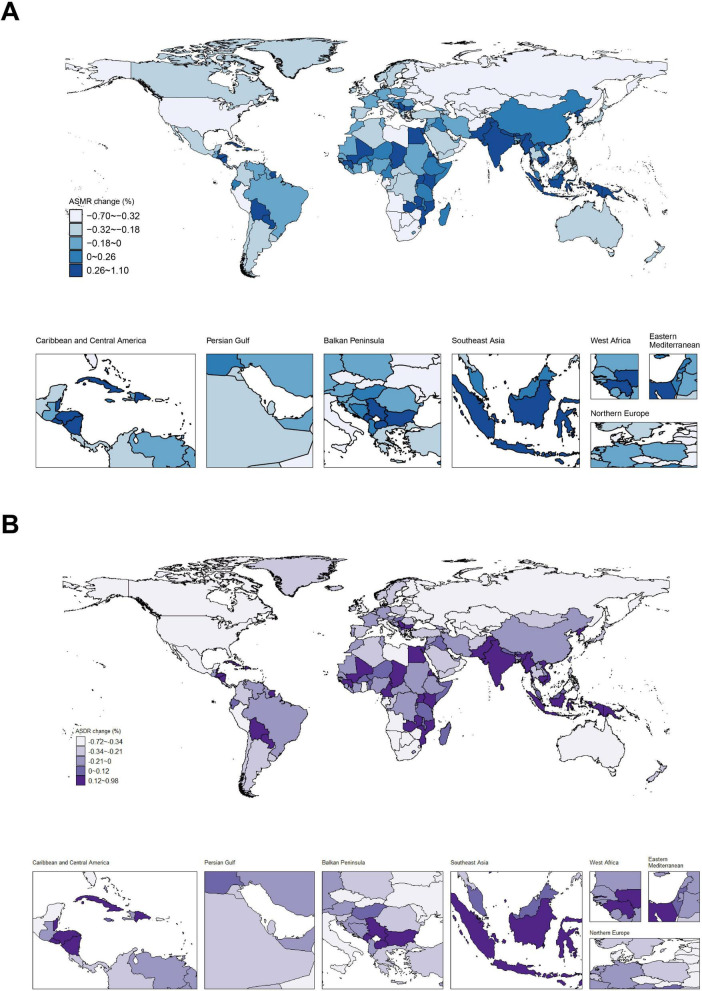
Distribution of ASMR and ASDR in nickel-associated lung cancer from 1990 to 2019. **(A)** Annual trend of ASMR in nickel-associated lung cancer; **(B)** Annual trend of ASDR in nickel-associated lung cancer.

From the perspective of ASMR for nickel-associated lung cancer, countries such as India, Pakistan, and Egypt have shown the largest annual average increase, ranging from 0.26 to 1.10%. Conversely, the United States, Russia, and South Africa experienced the largest annual average declines in this regard, with percentages ranging from 0.32 to 0.70% (Figure 5A). In terms of ASDR for nickel-associated lung cancer, Indonesia, Egypt, Serbia and other countries demonstrated the largest annual average increase at a range of 0.12–0.98%. On the other hand, countries such as the United States, Canada and Russia experienced significant annual average declines in ASDR of nickel-associated lung cancer at a range of 0.34 to 0.72% (Figure 5B). Overall trends indicate an upward trend in African and Southeast Asian countries while European and East Asian countries show a downward trend for nickel-associated lung cancer rates.

### 3.4 Age and gender distribution of the burden of lung cancer attributable to nickel exposure

**FIGURE 6:**
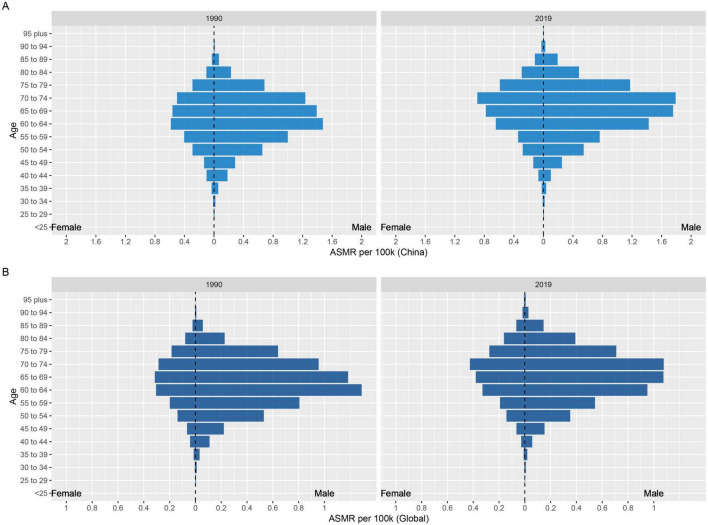
Age and sex-specific distribution of ASMR for nickel-associated lung cancer in 1990 and 2019. **(A)** In China; **(B)** In worldwide.

**FIGURE 7:**
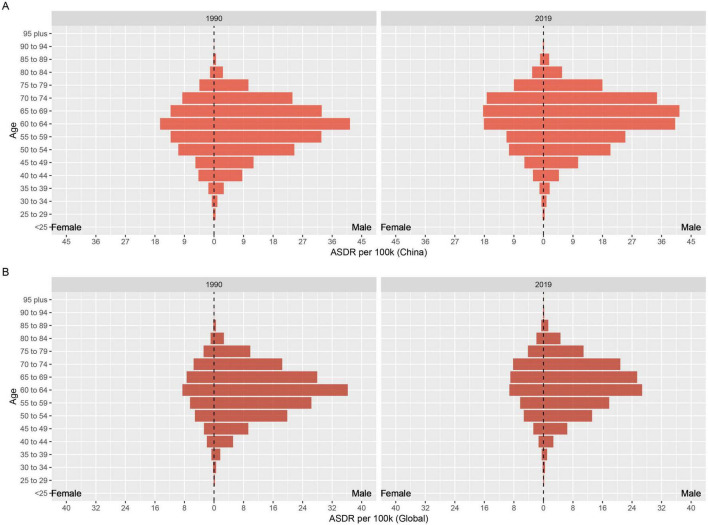
Age and sex-specific distribution of ASDR for nickel-associated lung cancer in 1990 and 2019. **(A)** In China; **(B)** In worldwide.

In the analysis of the burden of nickel-associated lung cancer by sex, it was observed that the ASMR and ASDR of nickel-associated lung cancer in China and the global population exhibited a pattern of initially increasing and then decreasing with advancing age, with higher rates in men than in women. The ASMR of nickel-associated lung cancer in Chinese men and women peaked in the 60–64 age group in 1990 and in the 70–74 age group in 2019 (Tables 3, 4 and Figure 6). In the global population, the ASMR of nickel-associated lung cancer reached its zenith in 1990 among women aged 65–69 and men aged 60–64. However, by 2019, both men and women showed a peak ASMR in the 70–74 age group (Tables 3, 4 and Figure 6). Similarly, the ASDR of nickel-associated lung cancer among Chinese men and women also peaked between the ages of sixty to sixty-four during both time periods (Tables 3, 4 and Figure 7); while globally, the ASDR reached its pinnacle at ages between sixty to sixty-four years old during both time periods (Tables 3, 4 and Figure 7).

**TABLE 3:** The age distribution on ASRs of lung cancer attributable to nickel in China, 1990.

	China				Global			
**Age**	**ASMR (×1/10^5^)**		**ASDR (×1/10^5^)**		**ASMR (×1/10^5^)**		**ASDR (×1/10^5^)**	
	**Male**	**Female**	**Male**	**Female**	**Male**	**Female**	**Male**	**Female**
< 25	0.000	0.000	0.000	0.000	0.000	0.000	0.000	0.000
25∼29	0.008	0.005	0.470	0.302	0.004	0.002	0.244	0.124
30∼34	0.018	0.012	1.030	0.671	0.010	0.005	0.572	0.268
35∼39	0.057	0.033	2.982	1.710	0.033	0.014	1.692	0.711
40∼44	0.183	0.101	8.615	4.746	0.110	0.041	5.170	1.937
45∼49	0.286	0.135	12.048	5.674	0.220	0.065	9.293	2.730
50∼54	0.655	0.291	24.485	10.885	0.531	0.138	19.855	5.177
55∼59	1.000	0.404	32.720	13.225	0.806	0.199	26.384	6.502
60∼64	1.475	0.585	41.441	16.438	1.289	0.305	36.238	8.563
65∼69	1.392	0.562	32.823	13.242	1.183	0.314	27.968	7.420
70∼74	1.237	0.501	23.907	9.661	0.955	0.286	18.485	5.518
75∼79	0.684	0.292	10.491	4.458	0.641	0.185	9.832	2.823
80∼84	0.230	0.103	2.718	1.220	0.227	0.079	2.683	0.929
85∼89	0.067	0.029	0.608	0.262	0.058	0.022	0.523	0.201
90∼94	0.010	0.004	0.074	0.030	0.007	0.003	0.052	0.024
≥ 95	0.001	0.000	0.004	0.003	0.001	0.001	0.007	0.004
Total	0.246	0.105	7.385	3.210	0.201	0.054	5.909	1.603

**TABLE 4:** The age distribution on ASRs of lung cancer attributable to nickel in China, 2019.

	China				Global			
**Age**	**ASMR (×1/10^5^)**		**ASDR (×1/10^5^)**		**ASMR (×1/10^5^)**		**ASDR (×1/10^5^)**	
	**Male**	**Female**	**Male**	**Female**	**Male**	**Female**	**Male**	**Female**
< 25	0.000	0.000	0.000	0.000	0.000	0.000	0.000	0.000
25∼29	0.006	0.004	0.377	0.253	0.003	0.002	0.186	0.095
30∼34	0.016	0.011	0.929	0.605	0.008	0.004	0.439	0.227
35∼39	0.037	0.023	1.899	1.202	0.019	0.009	0.996	0.474
40∼44	0.100	0.068	4.719	3.198	0.057	0.028	2.693	1.320
45∼49	0.250	0.137	10.576	5.796	0.153	0.065	6.456	2.738
50∼54	0.545	0.280	20.423	10.506	0.352	0.142	13.179	5.323
55∼59	0.762	0.344	24.965	11.256	0.544	0.193	17.817	6.311
60∼64	1.426	0.645	40.143	18.154	0.950	0.328	26.737	9.231
65∼69	1.756	0.781	41.448	18.399	1.074	0.380	25.391	8.966
70∼74	1.791	0.896	34.612	17.288	1.076	0.425	20.829	8.223
75∼79	1.174	0.591	17.987	9.003	0.709	0.275	10.866	4.202
80∼84	0.482	0.294	5.670	3.450	0.392	0.162	4.619	1.899
85∼89	0.192	0.116	1.743	1.041	0.145	0.066	1.316	0.591
90∼94	0.025	0.027	0.181	0.191	0.028	0.018	0.197	0.123
≥ 95	0.002	0.005	0.013	0.026	0.007	0.006	0.035	0.033
Total	0.255	0.124	7.054	3.468	0.163	0.061	4.509	1.711

### 3.5 Prediction of the burden of lung cancer attributable to nickel exposure in the next 15 years

**FIGURE 8:**
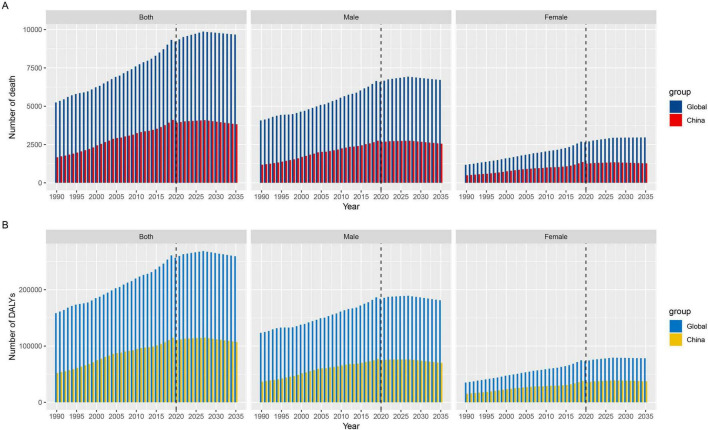
Projected trends in nickel-associated lung cancer mortality and DALY by sex in China and globally from 1990 to 2035. **(A)** Number of death; **(B)** Number of DALYs.

**FIGURE 9:**
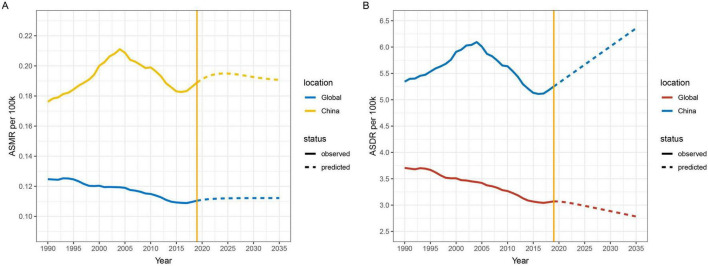
Projected trends in nickel-associated ASMR and ASDR by sex in China and globally from 1990 to 2035. **(A)** ASMR; **(B)** ASDR.

This study is based on the Nordpred model to predict the ASMR and ASDR of nickel-associated lung cancer in China and globally from 2020 to 2035. The results indicate that deaths related to nickel-associated lung cancer in China and globally are expected to reach their peak in 2027, with 4,087 and 9,874 cases, respectively (Table 5 and Figure 8A). The DALY of nickel-associated lung cancer in both China and worldwide shows a pattern of initial increase followed by decrease, reaching its peak in 2027 at 114,942 person-years and 268,731 person-years, respectively (Table 5 and Figure 8B). Table 5 and Figure 9A demonstrate that the ASMR of nickel-associated lung cancer in China exhibits an initial increase followed by decrease. Globally, the ASMR for nickel-associated lung cancer shows an initial increase followed by stabilization. Table 5 and Figure 9B show that the ASDR of nickel-associated lung cancer in China demonstrates an increasing trend; however, globally it shows a decreasing trend.

**TABLE 5:** Death and DALY of lung cancer attributable to nickel predicted by Norpred model from 2020 to 2035.

Year	Death cases		ASMR (×1/10^5^)		DALY numbers		ASDR (×1/10^5^)	
	**China**	**Global**	**China**	**Global**	**China**	**Global**	**China**	**Global**
2020	3,923	9,221	0.191	0.111	110,818	257,003	5.321	3.069
2021	3,967	9,369	0.193	0.111	111,976	260,106	5.390	3.058
2022	4,011	9,518	0.194	0.112	113,134	263,210	5.459	3.042
2023	4,026	9,589	0.195	0.112	113,496	264,314	5.528	3.025
2024	4,041	9,660	0.195	0.112	113,857	265,418	5.597	3.006
2025	4,056	9,731	0.195	0.112	114,219	266,523	5.666	2.986
2026	4,072	9,803	0.195	0.112	114,581	267,627	5.735	2.966
2027	4,087	9,874	0.194	0.112	114,942	268,731	5.804	2.946
2028	4,054	9,850	0.194	0.112	113,984	267,591	5.874	2.926
2029	4,021	9,826	0.193	0.112	113,027	266,451	5.943	2.905
2030	3,989	9,802	0.193	0.112	112,069	265,311	6.012	2.885
2031	3,956	9,778	0.192	0.112	111,111	264,172	6.081	2.865
2032	3,923	9,754	0.192	0.112	110,153	263,032	6.150	2.844
2033	3,890	9,729	0.191	0.112	109,195	261,892	6.219	2.824
2034	3,858	9,705	0.191	0.112	108,238	260,752	6.288	2.804
2035	3,825	9,681	0.191	0.112	107,280	259,612	6.357	2.784

## 4 Discussion

Based on the death toll, ASMR, DALY and other disease burden indicators in China and the world in GBD2019 database, this study analyzed and predicted the disease burden data and its changing trend of nickel-associated lung cancer. The normalized DALY, YLL and YLD rates of nickel-associated lung cancer in the Chinese population were calculated using the average age structure of the world population as the standard population, so as to form a comparable result with the global population. Compared with previous studies that mainly focused on the disease burden of lung cancer caused by risk factors such as tobacco and air pollution, this study focused on the disease burden of occupational nickel-exposed populations, paying more attention to occupational populations. It supplemented current neglected studies on the disease burden of nickel-associated lung cancer.

In 2019, the DALY of nickel-associated lung cancer in China accounted for 0.03% of the total disease burden, represented 43.91% of the global nickel-associated lung cancer disease burden (23). The disease burden of lung cancer caused by occupational nickel exposure is relatively small compared to other widely distributed risk factors for lung cancer (24, 25). However, it is important to note that our study found an increasing number of nickel-associated lung cancer deaths and ASDR in China and globally, with China experiencing a greater increase, the potential risk of developing lung cancer due to exposure to nickel cannot be overlooked, particularly within the context of China. On the contrary, there were no significant changes in the ASMR and ASDR of nickel-associated lung cancer, indicating there was no significant change in the impact of nickel on lung cancer within the population. The increasing burden of nickel-associated lung cancer is attributed to the aging age structure of patients.

The findings indicate that from 1990 to 2019, there was a consistent decrease in ASYLLR and an increase in YLD/YLL for both the Chinese and global populations affected by nickel-associated lung cancer. This suggests a reduction in the premature mortality impact of nickel-associated lung cancer, but an increase in the burden of disability. Furthermore, analysis of ASMR and ASDR for nickel-associated lung cancer in the Chinese population revealed a shift toward older age groups between 1990 and 2019. The reasons for this trend are twofold. Firstly, there is the acceleration of China’s population aging process (26, 27). Secondly, there has been an improvement in occupational health protection and medical care levels, as well as the implementation of effective three-level prevention measures (28–30). These factors have led to a reduction in disease mortality rates and an extension of patient survival times. However, a new challenge we face is how to improve the quality of life for patients.

Then, we projected the future trend of ASMR and ASDR in nickel-associated lung cancer in the Chinese population. It is anticipated that ASMR will initially increase and then decrease over the next 15 years, while ASDR is expected to show a continuous upward trend. At present, China’s new energy industry is developing rapidly, the scale of nickel industry is expanding, the occupational exposure of nickel and the general population exposure are increasing (31, 32), and China is in the aging process, the ASDR of nickel-associated lung cancer may highlight the rising trend. Compared with a general lung cancer risk analysis, this study focuses more on the harm caused by nickel as an occupational exposure factor to the population, which is also the key population exposed to nickel. With the robust development of the new energy industry in recent years, nickel has found extensive applications across various industrial sectors, including electroplating, battery manufacturing, and aerospace. This widespread utilization can be attributed to its exceptional corrosion resistance, physical strength, and distinctive ferromagnetic properties. Therefore, we contend that to effectively manage the disease burden of nickel-associated lung cancer, it is essential to prioritize the protection of occupational nickel-exposed populations. Additionally, it is crucial to address the impact of the aging age structure of patients on nickel-induced lung cancer. Finally, efforts should be made to mitigate the disease burden resulting from disability.

First and foremost, it is essential to implement effective primary prevention strategies aimed at reducing the incidence and mortality associated with nickel-associated lung cancer. Enhance the working environment and monitor nickel concentration levels in the workplace to ensure compliance with national safety standards. Implement advanced ventilation systems and utilize personal protective equipment in the workplace to effectively minimize workers’ direct exposure to nickel. In addition, it is essential to provide regular occupational health training for workers to enhance their awareness of the risks associated with nickel exposure, and user-friendly health education models specifically tailored for older workers should be developed. Second, enhance the routine occupational health assessments for workers exposed to nickel, and ensure effective implementation of secondary prevention measures. Enhance the monitoring and assessment of occupational health, and establish as well as improve personal health records for workers exposed to nickel. In particular, it is essential to increase the frequency of occupational health examinations for older workers. For individuals exhibiting mild symptoms or identified potential risks, early intervention measures such as job reassignment or a reduction in work intensity should be enacted. Third, it is essential to not only focus on the disease itself but also to consider the quality of life of patients with nickel-associated lung cancer, do a good job of tertiary prevention, and reduce the burden of disease. Timely identification of patients’ disabilities and mental health conditions, along with the provision of professional rehabilitation guidance and services for workers affected by disability or illness, is essential to assist them in regaining their physical function to the greatest extent possible. Enhance mental health support and offer psychological counseling to affected workers and their family members in order to alleviate the mental stress induced by the disease.

There are certain limitations to this study. First, all the data used in our study came from GBD 2019, which originated from various sources (33). Consequently, there may be inconsistencies and incompatibilities between these data, potentially leading to bias. Second, as a measure of disease burden, DALY is restricted to the patient group level and does not evaluate the burden caused by out-patient groups such as family, society, and the state. Finally, these data rely on the calculation of monitoring data, and there may be lags in estimating the current BOD.

## 5 Conclusion

In summary, although the DALY of nickel-associated lung cancer in the Chinese population is relatively low compared with other causes, it accounts for a high proportion globally. Additionally, the age structure of nickel-associated lung cancer patients shows an aging trend, and the ASDR in the Chinese population indicates a potential upward trend when projecting the disease burden of nickel-associated lung cancer over the next 15 years. Therefore, in the future, China should focus on occupational health prevention, strengthen protection for those in occupational contact with nickel, increase basic scientific research on nickel-associated lung cancer, pay attention to improving patients’ quality of life and mental health protection, and reduce the disease burden of nickel-related lung cancer within our population.

## Data Availability

The original contributions presented in this study are included in this article/supplementary material, further inquiries can be directed to the corresponding authors.

## Footnotes

1
http://ghdx.healthdata.org/gbd-results-tool
